# Combined analyses of kinship and *F*_ST_ suggest potential drivers of chaotic genetic patchiness in high gene-flow populations

**DOI:** 10.1111/mec.12341

**Published:** 2013-06-26

**Authors:** Matthew Iacchei, Tal Ben-Horin, Kimberly A Selkoe, Christopher E Bird, Francisco J García-Rodríguez, Robert J Toonen

**Affiliations:** * Hawai'i Institute of Marine Biology, School of Ocean and Earth Science and Technology, University of Hawai'i at MānoaKāne'ohe, HI, 96744, USA; † Department of Biology, University of Hawai'i at MānoaHonolulu, HI, 96822, USA; ‡ Bren School of Environmental Science and Management, University of California, Santa BarbaraSanta Barbara, CA, 93106, USA; § National Center for Ecological Analysis and SynthesisSanta Barbara, CA, 93101, USA; ¶ Department of Life Sciences, Texas A&M University – Corpus ChristiCorpus Christi, TX, 78412, USA; ** Instituto Politécnico Nacional, Centro Interdisciplinario de Ciencias Marinas, Colección IctiológicaLa Paz, Baja California Sur, 23096, México

**Keywords:** larval behaviour, marine connectivity, pelagic larval dispersal, phylogeography, population genetics, Southern California Bight, spiny lobster

## Abstract

We combine kinship estimates with traditional *F*-statistics to explain contemporary drivers of population genetic differentiation despite high gene flow. We investigate range-wide population genetic structure of the California spiny (or red rock) lobster (*Panulirus interruptus*) and find slight, but significant global population differentiation in mtDNA (Φ_ST_ = 0.006, *P* = 0.001; *D*_est_Chao_ = 0.025) and seven nuclear microsatellites (*F*_ST_ = 0.004, *P* < 0.001; *D*_est_Chao_ = 0.03), despite the species’ 240- to 330-day pelagic larval duration. Significant population structure does not correlate with distance between sampling locations, and pairwise *F*_ST_ between adjacent sites often exceeds that among geographically distant locations. This result would typically be interpreted as unexplainable, chaotic genetic patchiness. However, kinship levels differ significantly among sites (pseudo-*F*_16,988_ = 1.39, *P* = 0.001), and ten of 17 sample sites have significantly greater numbers of kin than expected by chance (*P* < 0.05). Moreover, a higher proportion of kin within sites strongly correlates with greater genetic differentiation among sites (*D*_est_Chao_, *R*^2^ = 0.66, *P* < 0.005). Sites with elevated mean kinship were geographically proximate to regions of high upwelling intensity (*R*^2^ = 0.41, *P* = 0.0009). These results indicate that *P. interruptus* does not maintain a single homogenous population, despite extreme dispersal potential. Instead, these lobsters appear to either have substantial localized recruitment or maintain planktonic larval cohesiveness whereby siblings more likely settle together than disperse across sites. More broadly, our results contribute to a growing number of studies showing that low *F*_ST_ and high family structure across populations can coexist, illuminating the foundations of cryptic genetic patterns and the nature of marine dispersal.

## Introduction

Determining the temporal and spatial scales of dispersal and gene flow across species’ ranges is essential for effective conservation and management. *F*-statistics (Wright 1943) and their analogues (e.g. Nei 1973; Weir & Cockerham 1984; Excoffier *et al*. 1992; Hedrick 1999) have been the workhorses in this regard for over 65 years. However, as both the number and diversity of genetic markers have increased, so has the demand for analyses that can complement fixation indices and extend our understanding of genetic data beyond the single marker, two-allele system pioneered by Wright (1943). Coalescent simulations (Kingman 1982) have emerged as the most informative techniques for distinguishing between historical and contemporary drivers of population differentiation (Hey & Wakeley 1997; Tavare *et al*. 1997; Rosenberg & Nordborg 2002; Rozas *et al*. 2003; Drummond *et al*. 2005; Hickerson *et al*. 2006; Hey & Nielsen 2007; Eldon & Wakeley 2009). By incorporating data from multiple nucleotide sequence-based markers, these equilibrium-independent analyses can isolate the effects of effective population size, demographic history, migration, mutation and drift summarized by *F*_ST_ (Hart & Marko 2010; Marko & Hart 2011, 2012). However, for fragment length data such as that generated by microsatellite markers, a number of alternative approaches have been advanced that can add insight into what is driving *F*_ST_ patterns (reviewed in Hedgecock *et al*. 2007; Lowe & Allendorf 2010).

One underutilized approach is the coupling of indirect metrics of gene flow (e.g. *F*-statistics, *D*_est_Chao_) with more direct measures such as kinship or parentage analyses (e.g. Loiselle *et al*. 1995; Selkoe *et al*. 2006; Buston *et al*. 2009; Christie *et al*. 2010; Palsbøll *et al*. 2010). Broadly speaking, kinship analyses provide an index of the relative relatedness of all genotyped individuals in a data set, and parentage is a distinct case of kinship whereby the most likely parents of individual juveniles are identified (Vekemans & Hardy 2004; Jones & Arden 2003; reviewed in Blouin 2003; Jones *et al*. 2010). Kinship coefficients (also known as coefficients of coancestry) are widely interpreted as the probability of identity by descent of the genes, but they are more properly defined as ‘ratios of differences of probabilities of identity in state’ (Hardy & Vekemans 2002, p. 23) from homologous genes sampled randomly from each pair of individuals (Hardy & Vekemans 2002; Rousset 2002; Blouin 2003; Vekemans & Hardy 2004).

By comparison, *F*-statistics and *D*_est_Chao_ are often blind to the relatedness of individuals; different population samples with the same kinship structure can have very different levels of genetic differentiation among them and vice versa. By assessing how alleles are shared among individuals, kinship analyses can elucidate which locations have comparatively little ongoing genetic exchange in situations where low *F*_ST_ values suggest high contemporary population connectivity. This clarification is important because such inferences can in fact be due to historically high migration rates, effective population sizes or measurement error (Hart & Marko 2010; Marko & Hart 2011, 2012; Faurby & Barber 2012).

Direct evidence of coancestry between individuals provides a particularly valuable complement to *F*-statistics when it is not possible to derive other independent estimates of demographic connectivity such as through the tagging and tracking of adults or larvae (e.g. Bellquist *et al*. 2008; Meyer *et al*. 2009; Cartamil *et al*. 2011; Carson *et al*. 2011; López-Duarte *et al*. 2012; reviewed in Lowe & Allendorf 2010). In marine systems, the majority of taxa have relatively sedentary adults, but a pelagic larval stage that persists in the water column from a few minutes to over a year (Thorson 1950; Strathmann 1987; McEdward 1995). These larvae are notoriously difficult to track directly (Levin 2006), but the time that larvae spend in the open ocean has led to the intuitive expectation that the majority of marine species have high levels of gene flow (Hedgecock *et al*. 2007). However, the preponderance of recent indirect genetic evidence, based mostly on *F*-statistics, indicates that there is generally a weak relationship between dispersal potential inferred from pelagic larval duration (PLD) and genetic structure (reviewed in Bradbury *et al*. 2008; Shanks 2009; Weersing & Toonen 2009; Riginos *et al*. 2011; Selkoe & Toonen 2011). Furthermore, it is generally overlooked that indirect gene flow via multigenerational stepping-stone dispersal at small scales can mimic direct gene flow across large scales (Puebla *et al*. 2012). The relatively few studies that have directly measured larval dispersal through larval tagging or parentage analyses have bolstered the claim that many larvae have limited dispersal and often recruit back to their region of origin (Jones *et al*. 2005; Gerlach *et al*. 2007; Planes *et al*. 2009; López-Duarte *et al*. 2012). Due to the nature of these direct (kinship/parentage) versus indirect (*F*-statistics) measures of population connectivity, it is possible that direct analyses may identify recruitment patterns that cannot be detected using traditional *F*-statistics (Waples & Gaggiotti 2006; Saenz-Agudelo *et al*. 2009; Palsbøll *et al*. 2010). For example, Christie *et al*. (2010) found little genetic differentiation (max *F*_ST_ = 0.0097) among populations of bicolor damselfish (*Stegastes partitus*) in Exuma Sound, Bahamas, but parentage analysis identified high levels of self-recruitment at two of the eleven sampled locations. The direct identification of parent–offspring pairs resulted in very different management advice for this species than interpretation based on the *F*_ST_ data alone.

To date, most marine kinship studies have understandably focused on parent–offspring identification in reef fishes with fairly short larval durations. Here, we show that kinship analyses can also be useful at the opposite end of the potential dispersal continuum: the California spiny, or red rock lobster, *Panulirus interruptus* (Randall 1840), spends at least the first 8 months of its life in the plankton, during which time it can presumably disperse across its entire geographic range. Species without barriers to dispersal are expected to exhibit no detectable neutral genetic population structure. Here, we use the California spiny lobster as a model to test the intuitive assumption of genetic homogeneity in species with extended PLD. We demonstrate the utility of individual-based estimates of genetic exchange in the interpretation of connectivity based on *F*-statistics.

## Methods

### Study system

The California spiny or red rock lobster, *Panulirus interruptus*, exhibits high site fidelity during its adult phase (Withy-Allen 2010), but has a two-phase pelagic larval stage with a total PLD of 8–11 months (Johnson 1956, 1960; Serfling & Ford 1975). The initial phyllosoma stage undergoes multiple moults to produce 11 vertically and geographically stratified stages in the pelagic environment (Johnson 1960; Pringle 1986). The 11th phyllosoma stage molts into the final puerulus stage, which settles into the lobster's preferred juvenile habitat. *Panulirus interruptus* can be found across a 1400-km Pacific coast range from Monterey Bay, CA (although very rare north of Point Concepcion) to Bahia Magdalena, Mexico. Throughout its geographic distribution, *P. interruptus* plays an important ecological role in structuring both kelp forest and intertidal communities (Tegner & Levin 1983; Robles 1987; Lafferty 2004). Spiny lobsters are also a valuable commercial and recreational fisheries species in both Mexico and the USA with a combined value of over $39 million from the most recent estimates (Chávez & Gorostieta 2010; Porzio 2012).

### Sample collection and DNA extraction

**Fig. 1:**
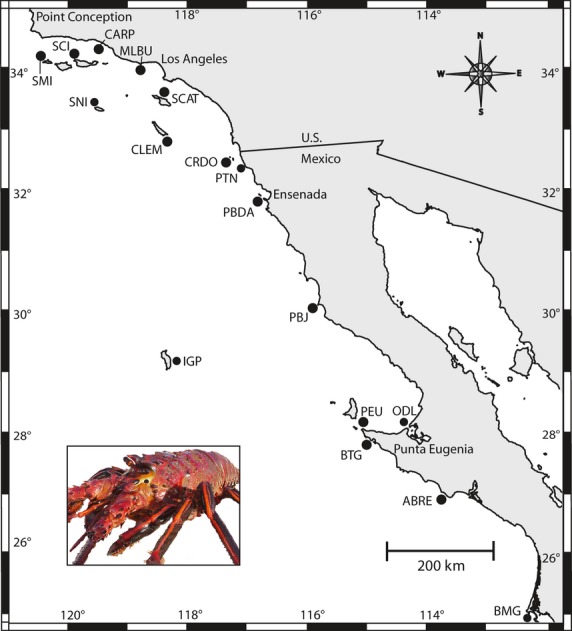
Map of lobster specimen collection locations in the Southern California Bight and Baja California, Mexico. CARP, Carpinteria, CA; SMI, San Miguel Island, CA; SCI, Santa Cruz Island, CA; MLBU, Malibu, CA; SCAT, Santa Catalina Island, CA; SNI, San Nicholas Island, CA; CLEM, San Clemente Island, CA; CRDO, Islas Coronados, Mx; PTN, Puerto Nuevo, Mx; PBDA, Punta Banda, Mx; PBJ, Punta Baja, Mx; IGP, Isla Guadalupe, Mx; ODL, Laguna Ojo de Liebre, Mx; PEU, Punta Eugenia, Mx; BTG, Bahia Tortugas, Mx; ABRE, Punta Abreojos, Mx; BMG, Bahia Magdalena, Mx. Photo credit: Patrick W. Robinson.

We collected tissue samples from 1102 *P. interruptus* individuals across 17 sites located throughout the entire Pacific coastal range from Point Conception, CA, in the north to Bahia Magdalena (BMG), Baja California Sur, Mexico, in the south (Fig. 1, Table 1). Samples were collected nonlethally by removing a small piece of an antenna or a leg segment from each lobster. Lobsters were either captured by hand while Scuba diving or obtained from commercial fishermen. Tissue samples were preserved in 95% ethanol and stored at room temperature until extracted. DNA was isolated using a standard salting-out protocol (Sunnucks & Hales 1996), a rapid-boil technique (Valsecchi 1998) or DNeasy Animal Tissue kits (Qiagen, Inc., Valencia, CA, USA).

**Table 1:** Summary statistics for *Panulirus interruptus* listed from northernmost to southernmost collection sites: total number of individuals sequenced for seven microsatellites (*N*, nDNA) and mtDNA cytochrome *c* oxidase subunit I (*N*, mtDNA)

Collection site (abbreviation)	*N*		*h*	*h*_*eff*_	π	AR	*A*_eff_	*H*_o_	*H*_e_

	nDNA	mtDNA							
Carpinteria (CARP)	74	57	0.94	16.67	0.011	18.46	15.98	0.83	0.89
San Miguel Island (SMI)	76	60	0.93	14.29	0.007	18.11	14.59	0.81	0.88
Santa Cruz Island (SCI)	54	53	0.92	12.50	0.005	18.48	15.51	0.84	0.89
Malibu (MLBU)	71	68	0.93	14.29	0.018	17.86	15.37	0.87	0.90
Santa Catalina Island (SCAT)	81	56	0.94	16.68	0.008	17.46	14.74	0.83	0.88
San Nicholas Island (SNI)	38	36	0.94	16.67	0.006	16.66	12.22	0.76	0.86
San Clemente Island (CLEM)	25	63	0.92	12.50	0.008	18.14	12.37	0.87	0.89
Islas Coronados (CRDO)	63	61	0.94	16.67	0.012	18.42	15.78	0.84	0.89
Puerto Nuevo (PTN)	57	56	0.91	11.11	0.007	16.83	13.65	0.86	0.88
Punta Banda (PBDA)	47	38	0.94	16.67	0.009	16.19	12.05	0.78	0.88
Punta Baja (PBJ)	94	70	0.88	8.33	0.009	17.85	15.06	0.86	0.88
Isla Guadalupe (IGP)	69	79	0.91	11.11	0.011	17.20	14.02	0.82	0.88
Laguna Ojo de Liebre (ODL)	42	55	0.91	11.11	0.008	18.51	15.34	0.83	0.88
Punta Eugenia (PEU)	45	45	0.93	14.29	0.012	17.36	13.39	0.82	0.89
Bahia Tortugas (BTG)	40	47	0.93	14.29	0.012	16.83	12.66	0.81	0.88
Punta Abreojos (ABRE)	66	42	0.88	8.33	0.009	17.44	14.96	0.84	0.89
Bahia Magdalena (BMG)	47	45	0.95	20.00	0.012	17.92	15.01	0.86	0.88

For mtDNA: haplotype diversity (*h*), effective number of haplotypes (*h*_eff_) and nucleotide diversity (π). For nDNA microsatellites: rarefied allelic richness (AR), effective number of alleles (*A*_eff_), observed (*H*_o_) and expected (*H*_e_) heterozygosity.

### Mitochondrial DNA (mtDNA)

We amplified a 494-bp fragment of cytochrome *c* oxidase subunit I gene (COI) using species-specific primers PintCOI-F (5′-GCTTGAGCTGGAATGGTAGG-3′) and PintCOI-R (5′-CACTTCCTTCTTTGATCCC-3′), which were designed from GenBank sequence #AF339460 (Ptacek *et al*. 2001) using primer3 (Rozen & Skaletsky 2000). Polymerase chain reactions (PCRs) for each sample were performed in a 20-μl reaction volume containing 10 μL of 2× Biomix Red (Bioline, Taunton, MA, USA), 0.125 μm each of forward and reverse primer, 5–50 ng of genomic DNA and 0.75× bovine serum albumin. PCR was carried out on a Bio-Rad Mycycler Thermal Cycler (Bio-Rad Laboratories Hercules, CA, USA), with an initial denaturation step of 95 °C for 4 min, 35 cycles of denaturation (95 °C for 30 s), annealing (56 °C for 30 s) and extension (72 °C for 30 s), followed by a final extension step of 72 °C for 10 min. PCR products were treated with 0.75 units of Exonuclease I and 0.5 units of Fast Alkaline Phosphatase (ExoFAP; Thermo Fisher Scientific, Waltham, MA, USA) per 7.5 μL of PCR product and incubated at 37 °C for 60 min, followed by deactivation at 85 °C for 10 min. Purified DNA fragments were sequenced in the forward direction with fluorescently labelled dideoxy terminators either on an ABI 3730XL capillary sequencer (Applied Biosystems, Foster City, CA, USA) by the Advanced Studies of Genomics, Proteomics and Bioinformatics (ASGPB) Center at the University of Hawai'i at Mānoa or an ABI 3130XL Genetic Analyzer (Applied Biosystems) at the Hawai'i Institute of Marine Biology EPSCoR Sequencing Facility. Unique sequences and sequences with ambiguous nucleotide calls were also sequenced in reverse to confirm sequence identity. Sequences were edited, aligned and trimmed to a uniform size using sequencher 4.8b (GeneCodes Corporation, Ann Arbor, MI, USA). The alignment did not contain any indels or frameshift mutations.

We calculated nucleotide (π) and haplotype diversity (*h*) for each sampling site as described in Nei (1987) using arlequin 3.5 (Excoffier *et al*. 2005). To visualize relationships between individual sequences, we constructed a median-joining network (Bandelt *et al*. 1999) using network 4.6.0.0 (http://www.Fluxus-engineering.com/network_terms.htm). We investigated population structure using an analysis of molecular variance (amova) as implemented in arlequin. We used an analogue of Wright's *F*_ST_ (Φ_ST_), which incorporates a model of sequence evolution, for both our complete data set and pairwise population comparisons (Weir & Cockerham 1984; Excoffier *et al*. 1992). Using jModelTest2 (Guindon & Gascuel 2003; Darriba *et al*. 2012), we determined that the Tamura & Nei (1993) with a Ti/Tv ratio of 11.2 and gamma parameter of 2.1 was the most appropriate model of sequence evolution implemented in arlequin. Global Φ_ST_ and each pairwise population Φ_ST_ were tested for significance with 100,000 permutations. Due to the high heterozygosity in cytochrome *c* oxidase subunit I gene (COI) in *P. interruptus*, we also calculated *D*_est_Chao_ as an absolute measure of differentiation between sites. The magnitude of *F*_ST_ is inversely proportional to heterozygosity (Hedrick 2005; Meirmans 2006; Jost 2008), while *D*_est_Chao_ is less susceptible to biases caused by genetic diversity (Bird *et al*. 2011). For mtDNA, *D*_est_Chao_ (Jost 2008) was calculated with spade (Chao & Shen 2009). Mantel tests using linearized *F*_ST_ [*F*_ST_/(1 − *F*_ST_)] and the natural log of Euclidean distance (Rousset 1997) were conducted in genodive 2.0b20 (Meirmans & Van Tienderen 2004) to test for isolation by distance (IBD) among all sample sites, as well as subsets of sites across the range (California sites, Mexico sites, all island sites, all mainland/continental sites).

### Microsatellite DNA

Seven previously developed microsatellites (A5, A102r, A110, Pin29L, Pin189, Pin10 and Pin244) (Ben-Horin *et al*. 2009) were amplified by PCR in a 10-μL reaction volume containing 1× GoTaq Reaction Buffer (with 1.5 mm MgCl_2_ pH 8.5; Promega Corp., Madison, WI, USA), 2 μm total dNTPs, 0.1 U GoTaq polymerase (Promega Corp.), 6 μm each of forward and reverse primer and 20 ng of genomic DNA. PCR was carried out using a Bio-Rad DNA Engine Dyad Thermal Cycler with the following conditions: an initial denaturation step of 95 °C for 3 min, 35 cycles of denaturation (94 °C for 40 s), annealing at primer-specific annealing temperatures (for 40 s; see Table 1 in Ben-Horin *et al*. 2009) and extension (72 °C for 40 s) and a final extension step of 72 °C for 30 min. Forward primers were fluorescently labelled with WellRed D2, D3 or D4 dye (Beckman Coulter Inc., Fullerton, CA, USA; see Table 1 in Ben-Horin *et al*. 2009 for primer labels). Microsatellite PCR products were sized on a Beckman Coulter CEQ 8000 capillary sequencer with a 400-bp size standard (Beckman Coulter Inc.). Alleles were scored using a CEQ 8000 genetic analysis system (Beckman Coulter Inc.).

Microsatellite quality control [Hardy–Weinberg equilibrium (HWE), linkage equilibrium, scoring errors] followed Selkoe & Toonen (2006) as detailed in Ben-Horin *et al*. (2009), but expanded across all sample locations and loci. Additionally, null allele frequency was re-calculated with *ML-Relate* (Kalinowski *et al*. 2006) to enable significance testing. We calculated observed (*H*_o_) and expected (*H*_e_) heterozygosities at each location, as well as allelic richness (*A*), rarefied to 25 individuals, using the Excel Microsatellite Toolkit 3.1.1 (Park 2001) and fstat (Goudet 2001), respectively. We also calculated the effective number of alleles at each locus in GenoDive. Overall genetic structure was analysed using an amova framework (Excoffier *et al*. 1992) implemented in genodive assuming the infinite allele model, with *F*_ST_ equivalent to Weir & Cockerham's θ (1984). Consistent with mtDNA (COI), microsatellite allelic diversity was likewise high, so we calculated *D*_est_Chao_ (Jost 2008) in addition to *F*_ST_. GenoDive was used to calculate pairwise *F*_ST_ and *D*_est_Chao_ for all sampling location pairs, and *F*_ST_ was tested for significance using 100 000 permutations. Patterns of IBD were investigated for microsatellite data in GenoDive as described above for mtDNA. The program geste 2.0 (Foll & Gaggiotti 2006) was used to calculate local *F*_ST_, a site-specific metric of allelic differentiation that accounts for the nonindependence inherent in multiple comparisons (Balding & Nichols 1995; Hudson 1998).

### Kinship

In order to further examine the factors driving the observed *F*_ST_ and *D*_est_Chao_ patterns, we calculated kinship coefficients (Loiselle *et al*. 1995) for each pair of individuals in genodive. These coefficients are based on the probability of identity of two alleles for each pair of homologous genes compared between each pair of individuals. Kinship was estimated with respect to the allele frequencies for the full data set, so these coefficients provide an index of relative relatedness between each pair of individuals. In order to determine whether individuals collected at the same location were more closely related to each other than individuals collected at different locations, we conducted an amova on the kinship coefficients. This approach compared the variation in within-population kinship coefficients with the variation in among-population kinship coefficients using the permanova+ 1.0.2 software add-on as implemented in primer6 (Clarke & Warwick 2001), following Stat *et al*. (2011) and Padilla-Gamiño *et al*. (2012). Specifically, the kinship covariance matrix created in genodive was loaded into primer6 as a Correlation Resemblance Matrix. A simple one-way analysis of variance (termed permanova in primer6) was conducted with 10 000 unrestricted permutations of the raw data and type III sums of squared differences. Significance is determined by evaluating a pseudo-F value (Clarke & Warwick 2001) based on the F-distribution, which is not to be confused with Wright's *F*-statistics.

To investigate site-specific patterns in kinship, we counted the number of closely related individuals within each site where the kinship coefficient was greater than or equal to the equivalent of that expected for quarter-siblings (0.047). Following Selkoe *et al*.'s (2006) and Buston *et al*.'s (2009) analysis of relatedness approach, we binned our counts of closely related lobsters according to specific levels of kinship. We used the Loiselle *et al*.'s (1995) coancestry coefficients (full-sib = 0.25, half-sib = 0.125) to generate the following bins: ‘nearly identical’, 0.57 > *k* > 0.375; ‘full-sib’, 0.375 > *k* > 0.1875; ‘half-sib’, 0.1875 > *k* > 0.09375; and ‘quarter-sib’, 0.09375 > *k* > 0.047. These bounds represent the midpoints between the coancestry coefficients in Loiselle *et al*. (1995), and we use quarter-sib as a short-hand to represent half of the level of coancestry as half-sibs. The nearly identical bin represents comparisons above full-sibs and is based on our kinship coefficient distribution for comparisons of individuals to themselves. We tested multiple bin sizes and divisions and found our results to be robust to these changes. To test for an overabundance of closely related lobsters within sites, we implemented a permutation test (10 000 replicates) where the lobsters were randomly assigned to sites and the observed number of closely related individuals was compared to the null distribution for each site. We calculated the maximum-likelihood estimate of relatedness (*r*) in *ML-Relate*, following the scale for the index of relatedness (full-sibs, *r* = 0.5; half-sibs = 0.25) to be able to compare our kinship results with a relatedness index. We also tested the relationship of the mean pairwise *F*_ST_, mean pairwise *D*_est_Chao_ and local *F*_ST_ for each site with the proportion of closely related lobsters at that site (summed across all four sibship categories for the Loiselle *et al*. (1995) metric and across half-sibs and full-sibs in *ML-Relate*).

### Upwelling

We identified hotspots of genetic differentiation by calculating mean *D*_est_Chao_ for each sampling location. We interpolated these values beyond the specific sites we sampled using an inverse weighted distance (IWD) algorithm in the Spatial Analyst extension in arcgis 10. To test the hypothesis that upwelling is a potential driver of both increased kinship and, in turn, site-specific genetic structure for *P. interruptus* in this region, we overlaid on this map known areas of consistent upwelling in Baja California, Mexico (as identified by Zaytsev *et al*. (2003)). We tested the relationship between the mean kinship at a site and that site's closest distance to an area of persistent upwelling. For sites within the Southern California Bight (SCB), where there are no areas of persistent upwelling, either the distance to Point Conception or to the edge of the northernmost upwelling area in Baja California, Mexico, was used, whichever was shorter. The southernmost sites in the range were measured to an upwelling area just off of BMG, which was identified by Zaytsev *et al*. (2003), but is not included in our figure. Negative distances represent sites that are located within upwelling regions and are measured from their location to the nearest edge of the upwelling zone. Both kinship and upwelling regression analyses were performed in spss 17.0.

## Results

### Mitochondrial DNA (mtDNA)

We sequenced COI for 931 individuals across 17 sites, which yielded 238 haplotypes. Haplotype diversity (*h*) was high, ranging from 0.88 to 0.95 (8.3–20 effective haplotypes), with a mean of 0.92 (12.5 effective haplotypes). In contrast, nucleotide diversity (π) was relatively low, ranging from 0.005 to 0.018, with a mean of 0.010. The number of individuals sequenced (N), haplotype diversity (*h*), effective number of haplotypes (*h*_eff_) and nucleotide diversity (π) for each site are listed in Table 1.

**Fig. 2:**
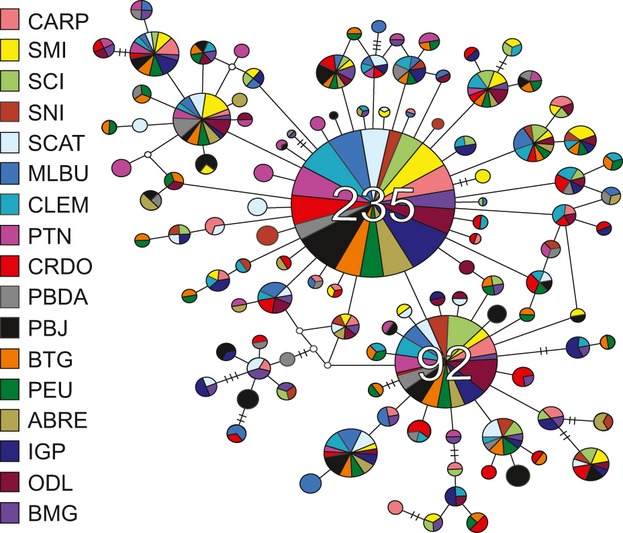
Median-joining network for *Panulirus interruptus* mtDNA, constructed using 454 base pairs of cytochrome *c* oxidase subunit I (COI) from each of 931 individuals in the program network 4.6.0.0. Each circle is a unique haplotype proportional in size to the number of individuals with that haplotype. The two largest circles represent 235 and 95 individuals. The smallest circle represents two individuals: there are 131 singletons in the data set, but these have been omitted for ease of visualization. A full, unedited network is included in the supplementary material (Fig. S3, Supporting information). Colours correspond to one of 17 locations where the individual haplotypes were found (see key, Fig. 1, Table 1). Lines connecting haplotypes represent a single base-pair difference between haplotypes, with crossing lines representing additional differences.

The median-joining network (Fig. 2) reveals two dominant haplotypes differing by one-nucleotide substitute and present at all sample sites. The most numerous haplotype was found in 235 individuals (25% of individuals sequenced), and the second most numerous haplotype was found in 92 individuals (10%). The haplotype network was characterized by a starburst pattern, with the majority of remaining haplotypes differentiated by one to two base pairs from the dominant haplotypes. Eighty haplotypes were represented in only two individuals and there were 131 singletons across all 17 sites; the removal of singletons did not impact the overall structure of the haplotype network (Fig. 2), so they were omitted for ease of visualization. A full, unedited network is included in Fig. S3 (Supporting information).

Global Φ_ST_ (0.006) was low, but significant (*P* = 0.001). Statistically significant pairwise Φ_ST_ values were 0.01–0.04, and in general, *D*_est_Chao_ values were an order of magnitude higher than Φ_ST_, both for global *D*_est_Chao_ (0.025) and for each of the pairwise comparisons, ranging up to 0.300 (Table 2). Among pairwise Φ_ST_ comparisons, 26 of 136 (19%, Table 2) were significant at *P* < 0.05; however, after correcting for the false discovery rate (Benjamini *et al*. 2006), no pairwise comparisons were significant (*P* < 0.00035, Table 2). There was no pattern of IBD (Wright 1943) at any scale in the data set. This was true whether we examined all sampling locations together, or for any of the specific subregions: all Mexico sampling locations, California locations (SCB), only islands and only continental sites (Table S1, Supporting information).

**Table 2:** *Panulirus interruptus* pairwise population structure results for (a) seven microsatellite loci and (b) a 494-bp fragment of the mtDNA cytochrome *c* oxidase subunit I region (COI)

	CARP	SMI	SCI	MLBU	SCAT	SNI	CLEM	CRDO	PTN	PBDA	PBJ	IGP	ODL	PEU	BTG	ABRE	BMG
*(a)*																	
CARP	—	0.035	0.011	0.013	0.016	0.030	0.028	−0.011	0.048	0.066	0.033	0.032	0.002	0.027	0.053	0.017	0.018
SMI	0.005*	—	0.013	0.030	0.026	0.031	0.016	0.019	0.063	0.073	0.035	0.003	−0.006	0.037	0.071	0.010	−0.008
SCI	0.001	0.002	—	0.008	0.028	0.048	0.014	0.006	0.043	0.049	0.025	0.006	−0.002	0.024	0.060	0.009	0.011
MLBU	0.001	0.004*	0.001	—	0.014	0.036	−0.005	−0.005	0.015	0.067	0.023	0.002	0.005	0.018	0.042	0	0.012
SCAT	0.002	0.004*	0.004*	0.002	—	−0.001	0.026	−0.005	0.041	0.089	0.040	0.029	0.007	0.034	0.029	0.013	0.023
SNI	0.004^‡^	0.005^‡^	0.007*	0.005^‡^	0	—	0.038	0.021	0.052	0.093	0.054	0.032	0.011	0.027	0.030	0.032	0.017
CLEM	0.003	0.002	0.002	−0.001	0.003	0.005	—	−0.009	0.056	0.073	0.035	0.010	−0.012	0.002	0.073	0.019	−0.005
CRDO	−0.001	0.002^‡^	0.001	−0.001	−0.001	0.003	−0.001	—	0.028	0.059	0.013	0.001	−0.012	0.008	0.037	−0.005	0.003
PTN	0.006**	0.009**	0.005*	0.002	0.006**	0.007*	0.007^‡^	0.004^‡^	—	0.104	0.046	0.026	0.023	0.058	0.077	0.031	0.044
PBDA	0.008**	0.010**	0.006*	0.008**	0.012**	0.013**	0.009*	0.008**	0.014**	—	0.104	0.066	0.075	0.069	0.128	0.081	0.070
PBJ	0.004**	0.005**	0.003^‡^	0.003*	0.005**	0.008**	0.005^‡^	0.002	0.006**	0.014**	—	0.016	0.013	0.045	0.079	0.025	0.012
IGP	0.004*	0	0.001	0	0.004*	0.005^‡^	0.001	0	0.004^‡^	0.009**	0.002^‡^	—	−0.006	0.030	0.065	0.001	−0.002
ODL	0	−0.001	0	0.001	0.001	0.002	−0.002	−0.001	0.003	0.010**	0.002	−0.001	—	0.005	0.047	−0.021	−0.02
PEU	0.003	0.005*	0.003	0.002	0.004*	0.004	0	0.001	0.007**	0.009**	0.006**	0.004^‡^	0.001	—	0.019	0.023	0.025
BTG	0.007*	0.010**	0.008**	0.005*	0.004^‡^	0.004	0.009*	0.005^‡^	0.010**	0.017**	0.011**	0.009*	0.006^‡^	0.002	—	0.051	0.064
ABRE	0.002	0.001	0.001	0	0.002	0.005^‡^	0.002	−0.001	0.004*	0.011**	0.003*	0	−0.003	0.003	0.007*	—	0.003
BMG	0.002	−0.001	0.001	0.001	0.003^‡^	0.002	−0.001	0	0.006*	0.009**	0.002	0	−0.003	0.003^‡^	0.008**	0	—
*(b)*																	
CARP	—	0.003	0.016	0.018	0	0.118	0	0.067	0.004	0.005	0.081	0	0.015	0	0	0.039	0
SMI	0	—	0.124	0.022	0	0.214	0	0.032	0.020	0.011	0.057	0.008	0.058	0	0	0.003	0
SCI	−0.002	0.005	—	0.108	0.100	0	0.025	0.209	0.072	0.027	0.143	0.068	0	0.005	0	0.096	0.093
MLBU	0.009	0.008	0.015	—	0	0.189	0	0.033	0.037	0.033	0.072	0.024	0.055	0	0	0.016	0
SCAT	0.007	0.002	0.005	0.001	—	0.144	0	0.044	0.024	0	0.072	0	0.057	0	0	0.021	0
SNI	0.003	0.006	−0.002	0.013	0.002	—	0.117	0.300	0.174	0.068	0.253	0.164	0.054	0.081	0.070	0.190	0.171
CLEM	0.001	0.002	0.007	0.011	0.013	0.009	—	0	0	0	0.025	0	0	0	0	0	0
CRDO	−0.002	0.003	−0.001	0.006	0	0.002	0.002	—	0.080	0.135	0.090	0.038	0.165	0	0.017	0.06	0
PTN	0.018	0.003	0.024*	0.015	0.007	0.021	0.013	0.018	—	0.018	0.033	0	0.023	0	0	0	0.008
PBDA	0.001	0.003	0.005	0.007	−0.007	0.003	0.014	−0.005	0.013	—	0.126	0.042	0	0	0	0.031	0.015
PBJ	0.019	0.022	0.008	0.013	0.001	0.010	0.03*	0.008	0.034*	0.002	—	0.022	0.098	0.009	0.003	0	0.072
IGP	−0.003	0.001	−0.002	0.006	−0.003	−0.002	0.002	−0.003	0.012	−0.005	0.008	—	0.048	0	0	0	0
ODL	−0.002	−0.005	−0.005	0.002	−0.003	−0.001	−0.003	−0.002	0.004	−0.003	0.009	−0.005	—	0	0	0.027	0.073
PEU	0.002	0	0	0.005	0	0.004	−0.006	0.001	0.001	0.005	0.015	0.001	−0.007	—	0	0	0
BTG	0.003	0.002	−0.002	0.006	0	0.004	−0.004	0.001	0.005	0.005	0.012	0.001	−0.006	−0.021	—	0	0
ABRE	0.002	−0.002	0.008	0.004	0.004	0.001	−0.003	0.005	0.003	0.009	0.020	0.002	−0.005	−0.005	−0.003	—	0.023
BMG	0.018	0.025	0.014	0.022	0.006	0.014	0.043**	0.008	0.040*	−0.005	0.002	0.006	0.017	0.031*	0.029	0.035*	—

*F*_ST_ (ф_ST_ for COI) is below the diagonal, and Jost's *D*_est_Chao_ is above the diagonal. Shaded boxes indicate significant differences at *P* < 0.05 for both nDNA and mtDNA.

For nDNA, comparisons marked with *, ** or ^‡^ are significant after a false discovery correction is applied. *P < 0.01, **P < 0.0005. For mtDNA, no comparisons were significant after correcting for false discovery, although some were significant at *P* < 0.01 and 0.0005, and indicated as such.

CARP, Carpinteria, CA; SMI, San Miguel Island, CA; SCI, Santa Cruz Island, CA; MLBU, Malibu, CA; SCAT, Santa Catalina Island; SNI, San Nicholas Island, CA; CLEM, San Clemente Island, CA; CRDO, Islas Coronados, Mx; PTN, Puerto Nuevo, Mx; PBDA, Punta Banda, Mx; PBJ, Punta Baja, Mx; IGP, Isla Guadalupe, Mx; ODL, Laguna Ojo de Liebre, Mx; PEU, Punta Eugenia, Mx; BTG, Bahia Tortugas, Mx; ABRE, Punta Abreojos, Mx; BMG, Bahia Magdalena, Mx.

### Microsatellite DNA

We scored 989 individuals across 17 sites for seven nuclear microsatellite loci, with five to 104 alleles per locus, which translated to between two and 23 effective alleles per locus (Table S2, Supporting information). The genotyping error rate, determined by re-genotyping 74 individuals at each locus, ranged from 0.0% to 4.1%, with an average of 2.3% overall (Table S2, Supporting information). We identified two pairs of individuals with identical genotypes and removed one individual of each pair from the data set in order to eliminate the possibility that the same individual was sampled two times. In both cases, identical genotypes were identified within a sampling site. The expected chances of observing true identical twins in this study ranged from 1 × 10^−42^ to 4.39 × 10^−26^, while our observed rate was substantially greater, 2 in 989 specimens.

Rarefied allelic richness was similar among sites and ranged from 16.19 to 18.51, while the effective number of alleles ranged from 12.05 to 15.98 (Table 1). Expected heterozygosity (*H*_e_) was between 0.86 and 0.90, while observed heterozygosity (*H*_o_) exhibited a slightly wider range from 0.76 to 0.87 (Table 1). Tests for linkage disequilibrium (LD) were significant in 20 of 349 comparisons (∼6%) after correcting for multiple tests, and there were no locus-specific patterns. The three sample sites with the highest percentage of LD comparisons correspond to the three sites with the highest levels of kinship, as reported below, suggesting kinship may be high enough at these sites to produce a signal of LD. In general, however, LD is a weak test of family structure, and we found no site-specific patterns across the rest of the sites in the study. There were significant deviations from HWE in 46 of 119 (∼39%) comparisons after correcting for multiple comparisons, but again no sample-specific patterns were observed. micro-checker 2.2.3 (Van Oosterhout *et al*. 2004) found no evidence of scoring errors due to large allele dropout or stutter; however, six of seven markers showed patterns consistent with null alleles, which are the likely cause of the deviations from HWE (see Ben-Horin *et al*. 2009). Although the frequencies of these null alleles are low (1.20–6.63% across loci, Table S2, Supporting information), we wanted to be sure they would not affect our results. To test for the impact of null alleles on our results, we used FreeNA (Chapuis & Estoup 2007) to generate alleles for the data set where nulls were expected, re-analysed the data, and our subsequent results and conclusions remained the same. Therefore, we present only analyses with the full original data set.

Significant partitioning of the samples among locations was detected in the microsatellite loci with both a global fixation test (*F*_ST_ = 0.004, *P* < 0.001) and a global genetic differentiation test (*D*_est_Chao_ = 0.03, *P* < 0.0005). Global partitioning was also detected in analyses of individual loci, with a significant global *F*_ST_ (0.002–0.011, Table S2, Supporting information) and global *D*_est_Chao_ (0.030–0.114) at each individual locus, except A110.

We ran pairwise *F*_ST_ and *D*_est_Chao_ comparisons for each marker individually, as well as jackknifing across markers, and found the results were fairly consistent throughout these comparisons. Significant pairwise *F*_ST_ comparisons among sampling sites using the microsatellite loci were fairly low, ranging from 0.002 to 0.015, but 71 of 136 comparisons (52%, Table 2) remained significant after correcting for the false discovery rate (Benjamini *et al*. 2006). As in our mtDNA results, pairwise *D*_est_Chao_ comparisons generally were an order of magnitude higher than each respective pairwise *F*_ST_ comparison and ranged from −0.021 to 0.128 (Table 2). The higher magnitude of *D*_est_Chao_ compared *F*_ST_ to matches the expectation when heterozygosity is ∼0.9, as it is in this study (Bird *et al*. 2011).

The sites with the highest mean pairwise *F*_ST_ and *D*_est_Chao_ values were in the northern and central Baja California, Mexico region [Puerto Nuevo (PTN), Punta Banda (PBDA), Punta Baja and Bahia Tortugas (BTG)]. These sites also stood out by having the highest proportion of significant differences when compared to the other sites. Three of these four sites also had the highest local *F*_ST_ values (PBDA, BTG and PTN). There was no pattern of IBD in the data set, whether we examined all sampling locations together, or for any of the specific subregions, as described for the mtDNA results (Table S1, Supporting information).

### Kinship

**Fig. 3:**
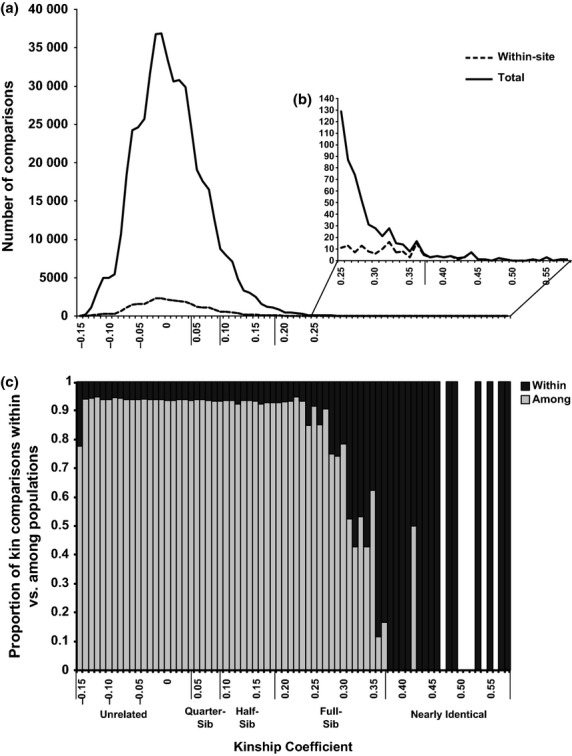
(a) Line graph depicting the total number of kinship (*k*) pairwise comparisons for each 0.01 bin of kinship (solid line, total *N* = 457,652) and the number of within-site kinship comparisons (dashed line, total *N* = 30,914). (b) Inset of (a), depicting the total number of kinship comparisons (solid line) and the number of within-site comparisons (dashed line) from 0.25 to 0.57 on a separate *y*-axis that ranges from 0 to 140 comparisons. The majority of these kinship comparisons are between individuals sampled at the same location. (c) Distribution of kinship coefficients divided into 0.01 bins and coloured by the proportion of within-site (dark grey) versus among-site (light grey) comparisons within each 0.01 division. White bars represent levels of kinship that were not found in the data set. Bars on the *x*-axis represent the divisions between unrelated and related individuals and between each of the four kinship categories we analysed: ‘quarter-sib’, 0.047 < *k* < 0.09375; ‘half-sib’, 0.09375 < *k* < 0.1875; ‘full-sib’, 0.1875 < *k* < 0.375; and ‘nearly identical’, 0.375 < *k* < 0.57.

**Fig. 4:**
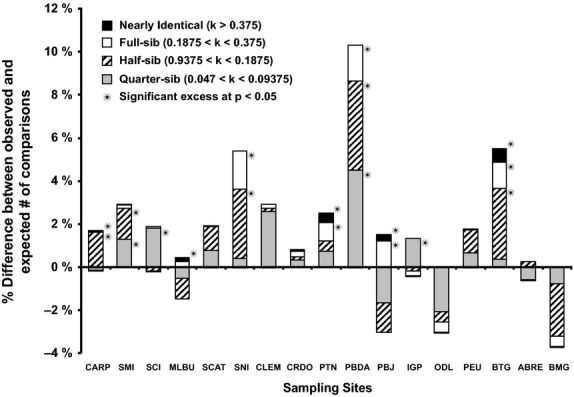
Histogram of the proportion of kinship observed for each site that is in excess of expected levels due to chance in four kinship categories: ‘quarter-sib’, 0.047 < *k* < 0.09375; ‘half-sib’, 0.09375 < *k* < 0.1875; ‘full-sib’, 0.1875 < *k* < 0.375; and ‘nearly identical’, 0.375 < *k* < 0.57. Asterisks indicate significant (*P* < 0.05) differences between the observed and expected proportion of kinship comparisons within that category at that site. Expected kinship levels were constructed using 10 000 permutations of all kinship values across all sites without replacement to generate the distribution of kinship values that should occur if individuals were randomly distributed among sites. The nearly identical bin represents comparisons above full-sibs and is based on our kinship coefficient distribution for comparisons of individuals to themselves. Counts of the actual number of pairwise comparisons at each site that fell within each kinship category are listed in Table S3.

**Fig. 5:**
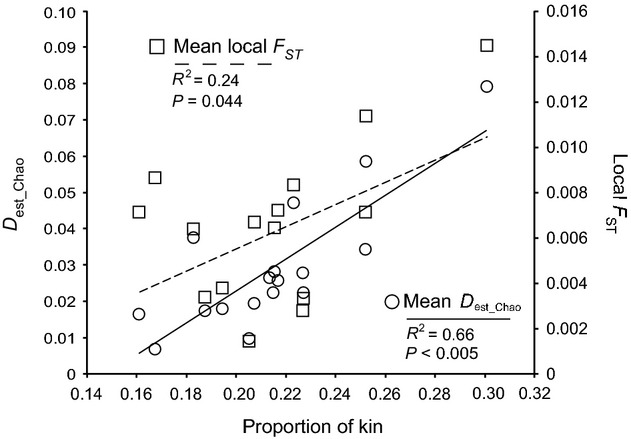
Linear regression of mean pairwise *D*_est_Chao_ (circles, solid line) and local *F*_ST_ (squares, dashed line) for seven microsatellite loci. Both metrics were regressed on the proportion of within-site kinship comparisons at each site that are significantly (*P* < 0.05) greater than *k* = 0.047 (the lower boundary of the four designated kinship categories).

Following the exclusion of lobster specimens with identical genotypes, as well as self-comparisons, kinship coefficients ranged from −0.155 to 0.57 (Fig. 3). The overall mean kinship, which is expected to be zero, was −0.000025 ± 0.00008SE. There was a disparity, however, between the within-site mean kinship (0.003 ± 0.0004) and the among-site mean kinship (−0.0002 ± 0.00009) (Fig. 3). Mean within-site maximum-likelihood estimates of relatedness (*r*, from *ML-Relate*) ranged from 0.034 to 0.057 (Table S3, Supporting information) and were significantly correlated with mean kinship values (*k*) at each site (Table S3, Supporting information, Pearson's *r* = 0.92, *P* < 0.0005). Kinship coefficients were significantly greater for within-site than for among-site comparisons (pseudo-*F*_16,988_ = 1.39, *P* = 0.001). In total, 10 of 17 sites had significantly greater numbers of closely related pairs of individuals than expected by chance (*P* < 0.05) in at least one of four kinship categories: five sites had an excess of individuals in the ‘nearly identical’ category (0.57 > *k* > 0.375), five sites had an excess in the ‘full-sib’ group (0.375 > *k* > 0.1875), five sites had an excess in the ‘half-sib’ category (0.1875 > *k* > 0.09375), and four sites had an excess in the ‘quarter-sib’ bin (0.09375 > *k* > 0.047) (Fig. 4, Table S3, Supporting information). The proportion of kin in each site was significantly related to mean pairwise *F*_ST_ (*R*^2^ = 0.669; *F*_1,16_ = 30.333, *P* < 0.0005) and *D*_est_Chao_ (*R*^2^ = 0.658; *F*_1,16_ = 28.885, *P* < 0.0005; Fig. 5) for each site as well as to local *F*_ST_ (*R*^2^ = 0.243; *F*_1,16_ = 4.825, *P* = 0.044; Fig. 5). These findings are consistent across kinship classes. When we removed the lowest level of kinship (examining only kinship levels equivalent to ‘nearly identical’, ‘full-sib’ and ‘half-sib’), the relationship of both *F*_ST_ (*R*^2^ = 0.672; *F*_1,16_ = 30.741, *P* < 0.0005) and *D*_est_Chao_ (*R*^2^ = 0.658; *F*_1,16_ = 28.887, *P* < 0.0005) with the proportion of kin stayed approximately the same, while the relationship of local *F*_ST_ with the proportion of kin strengthened (*R*^2^ = 0.303; *F*_1,16_ = 6.526, *P* = 0.022). The relationships of mean pairwise *F*_ST_, *D*_est_Chao_ and local *F*_ST_ with the proportion of related individuals (*r*, full- and half-sibs) from *ML-Relate* were all significant and stronger than the relationships between these summary statistics and proportion of kin (Fig. S1, Supporting information).

### Upwelling

**Fig. 6:**
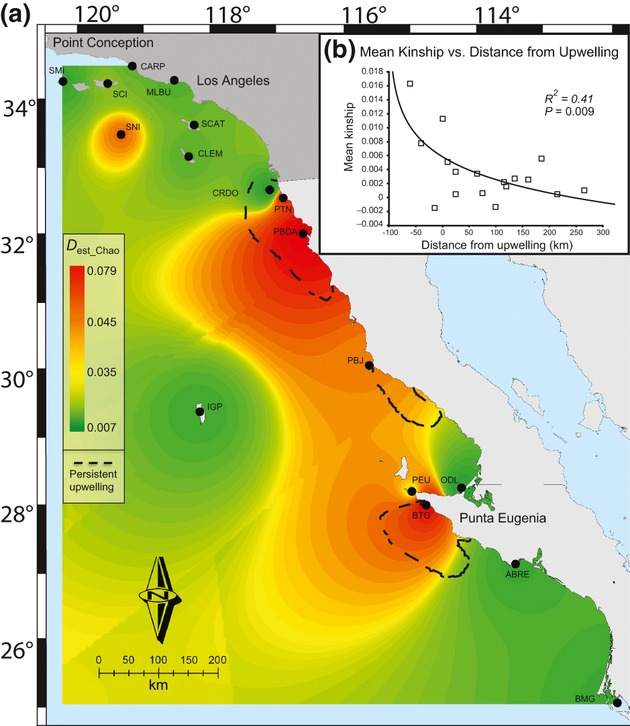
(a) Map of lobster specimen collection locations overlaid with an inverse weighted distance interpolation of mean *D*_est_Chao_ values at each site created using the Spatial Analyst extension in arcgis 10. Red coloration represents areas of greatest genetic differentiation, green represents areas of little genetic differentiation, and yellow and orange represent the graded values between these extremes. Mean kinship values at each site showed the same pattern as *D*_est_Chao_, with the highest kinship in red and lowest in green. Dashed lines circle areas of consistently high upwelling intensity (adapted from Zaytsev *et al*. 2003). There are also areas of high upwelling intensity at Point Conception, and one south of Bahia Magdalena that are not captured by this map. (b) Log-linear regression of mean kinship at each site on the distance (km) to the nearest edge of an area of high upwelling intensity [from (a)].

Regions containing persistent, strong upwelling regimes, both across seasons within a year and across all years from 1996 to 2002, were traced from fig. 14 in Zaytsev *et al*. (2003) and are depicted by dotted lines in Fig. 6a. Three of the four upwelling regions overlap the geographic range of our study and contain four of our sampling locations: Islas Coronados, PTN, PBDA and BTG (Fig. 6a). Three of these four sites (PTN, PBDA and BTG) contain one of the highest four values for mean *D*_est_Chao_, mean local *F*_ST_ and mean kinship. The IWD function applied to this data extrapolates these site-specific metrics across the entire study area, including the unintended extrapolation of *D*_est_Chao_ values to ocean areas where adult lobsters do not live, but phyllosoma may be present. Along the Baja California coastal areas containing adult lobsters, all three of the areas with the highest genetic differentiation (*D*_est_Chao_) overlap the regions of strong upwelling intensity (Fig. 6a). The results for mean kinship and local *F*_ST_ were similar, although not shown. Across the geographic extent of our study, we see a significant relationship (*R*^2^ = 0.407; *F*_1,16_ = 10.296, *P* = 0.006) between mean kinship at each site and the closest distance between each site and the edge of an upwelling zone [(mean kinship + 1) = −0.005 ln (distance to upwelling + 100 km) + 1.028, Fig. 6b). This relationship holds for mean relatedness (*r*) versus distance from upwelling as well (Fig. S2, Supporting information).

## Discussion

There is accumulating evidence from multiple approaches that larvae rarely reach their full dispersal potential, resulting in a paradigm shift away from the perception that most marine populations are genetically homogenous across broad geographic scales (Jones *et al*. 1999; Swearer *et al*. 1999, 2002; Mora & Sale 2002; Grantham *et al*. 2003; Taylor & Hellberg 2003; Marko 2004; Cowen *et al*. 2006; Becker *et al*. 2007; López-Duarte *et al*. 2012). However, this evidence has come exclusively from species with short to modest larval periods (1–60 days). Even among species with modest PLD, there are just a few striking examples of broad populations with no genetic substructure across their full range. For example, reef fishes *Myripristis jacobis* in the Atlantic Ocean (Bowen *et al*. 2006), *Lutjanus kasmira* in the Central Pacific and Eastern Indian Oceans (Gaither *et al*. 2010) and *Acanthurus nigrofuscus* in the Pacific (Eble *et al*. 2011) all exhibit genetic homogeneity across thousands of kilometres (up to 12 000 km). Dispersal potential is assumed to be great in species with very long PLDs (>120 days), and population genetic surveys of such species to date have revealed little population structuring across broad geographic scales (Ovenden *et al*. 1992; Silberman *et al*. 1994; Thompson *et al*. 1996; Tolley *et al*. 2005; García-Rodríguez & Perez-Enriquez 2006; Inoue *et al*. 2007; Horne *et al*. 2008; Reece *et al*. 2011). When genetic discontinuities have been observed in species with long PLD, they have invariably corresponded with known biogeographic barriers, or oceanographic transitions (Palero *et al*. 2008; Babbucci *et al*. 2010; Chow *et al*. 2011).

In contrast to the intuitive expectation that *Panulirus interruptus*, with a minimum PLD of 240 days, would be genetically homogenous across its entire 1400-km range along the west coast of North America, we found slight, but significant genetic structuring among several sampling locations throughout Mexico and Southern California (Table 2). This finding contrasts with previous work that did not detect population structure in *P. interruptus* throughout Baja California, Mexico, using mtDNA RFLPs (García-Rodríguez & Perez-Enriquez 2006). Notably, lobsters do not exhibit a genetic break across Punta Eugenia, a faunal boundary for rocky intertidal species (Valentine 1966; Blanchette *et al*. 2008; Gaines *et al*. 2009) and a phylogenetic break for a number of coastal fishes (Bernardi *et al*. 2003). Nor does the overall pattern of genetic differentiation in *P. interruptus* correspond to the Northern, Central and Southern regional population subdivision within Baja predicted by Perez-Enriquez *et al*. (2001). Rather, genetically differentiated sites are nested within a greater area of undifferentiated sites (Fig. 6a). Specifically, some sites exhibit no genetic differentiation across the 1400-km species range, whereas other sites are differentiated from the majority of sampled sites, and there is no signal of IBD in either the mtDNA or the nuclear microsatellite markers across multiple spatial scales (Table S1, Supporting information). Similar patterns of genetic differentiation among proximate sites have been shown in species with shorter larval developmental periods (reviews by Larson & Julian 1999; Hedgecock *et al*. 2007; Riginos *et al*. 2011; Toonen & Grosberg 2011), but have not been reported for a species with such a long PLD as this one. Additionally, *P. interruptus* has equivalent or greater levels of genetic substructure than other species occurring in this same region (Selkoe *et al*. 2010), despite a PLD that is an order of magnitude higher.

The term ‘chaotic genetic patchiness’ was coined (Johnson & Black 1982, 1984) to describe ephemeral, finely spatio-temporal patterns of genetic structure generated by variation in the larval pool, recruitment and natural selection, which are counteracted in the long-term by dispersal and gene flow (Toonen & Grosberg 2011). Much of the difficulty in interpreting these unexpected patterns in genetic differentiation is due to the nature of *F*_ST_ as a summary statistic. Significant structure among populations may be a result of differences in effective population size (and corresponding genetic drift), demographic or colonization history, migration or some combination of these factors, especially for populations that may not have reached migration–drift equilibrium. Direct interpretation of summary statistics (*F*_ST_*, D*_est___Chao_) in the context of gene flow can be problematic (reviewed by Lowe & Allendorf 2010; Hart & Marko 2010; Marko & Hart 2011; Bird *et al*. 2011; Karl *et al*. 2012), especially in species such as *P. interruptus*, with highly fecund individuals and a potential for reproductive skew (Eldon & Wakeley 2009). For the many marine species with high fecundity and a type III survivorship curve, an independent test can help determine whether gene flow is the primary driver resulting in the observed population structure (Hart & Marko 2010; Lowe & Allendorf 2010).

Here, kinship (Loiselle *et al*. 1995) enriches our understanding of the drivers underlying significant differences in *F*_ST_ among sites. The pattern of chaotic genetic patchiness in *P. interruptus* evident in the *F*_ST_ and *D*_est___Chao_ analyses seems to be primarily a result of the nonrandom occurrence of closely related lobsters within sample sites. Across all sites, lobsters were more closely related within sites than between sites, and at the majority of sites, we found significantly greater than expected levels of kinship between adult lobsters (Fig. 4). Moreover, the proportion of kin found at each site accounts for the majority of the variation in the sites’ genetic differentiation: the most greatly differentiated sampling sites have the highest proportion of kin (Fig. 5).

One potential scenario that could generate high proportions of kin within sites is recruitment pulses of related individuals. The simplest explanation for this phenomenon is that larvae released together stay together throughout dispersal and recruitment (kin aggregation). However, this pattern would also result from extreme differential reproductive success among individuals, so that a recruiting cohort is entirely made up of offspring from only a few individuals (sweepstakes recruitment, Hedgecock 1986, 1994a,b). Sweepstakes recruitment could also generate the star-shaped pattern of our mtDNA haplotype network (Fig. 2, Fig. S3, Supporting information), although this pattern could also be indicative of a recent population expansion. Previous kinship analyses have detected high levels of relatedness within cohorts of larval recruits in both fishes (Planes *et al*. 2002; Pujolar *et al*. 2006; Selkoe *et al*. 2006; Buston *et al*. 2009; Bernardi *et al*. 2012) and invertebrates (Veliz *et al*. 2006), which supports both the hypothesis of kin aggregation throughout development and/or the hypothesis of sweepstakes reproduction. Unfortunately, we could not directly test these alternatives in *P. interruptus* because we did not have samples of new recruits. Nevertheless, given the size selectivity of lobster traps, and the intense fishing pressure for lobster depressing the age range (Iacchei *et al*. 2005; Kay & Wilson 2012), it is possible that our samples are largely made up of single year classes consisting of closely related individuals recruiting together by one of these aforementioned mechanisms. To our knowledge, this study is the first documented case of kin aggregation in the adult population of a marine species with planktonic larvae, although kin aggregation in recruits has been reported. Previous studies that have looked at only kin relationships among adults, rather than among cohorts of recruits, have found no evidence of kin aggregation in marine species (Avise & Shapiro 1986; Kolm *et al*. 2005; Buston *et al*. 2007; Palm *et al*. 2008; Andrews *et al*. 2010; Berry *et al*. 2012). Consequently, kin aggregation is generally assumed to be a transient phenomenon limited to newly settled recruits, with little detectable signal in adult populations due to multiple source populations of recruits, changes in reproductive success and differential juvenile mortality (Kordos & Burton 1993; Moberg & Burton 2000; Flowers *et al*. 2002; Planes *et al*. 2002; Selkoe *et al*. 2006; Buston *et al*. 2009).

High levels of within-site kinship could also be driven by a temporally stable pattern of self-recruitment, either through larval retention or through larval dispersal with subsequent recruitment back to the natal site. The prospect that larvae stay in the plankton for 240–330 days and return to settle near their site of release seems unlikely at first. However, the site-specific kinship patterns in our data match theoretical predictions for a species that has evolved a long PLD to avoid predation during the larval phase rather than to facilitate broad dispersal of larvae (Strathmann *et al*. 2002). The extended PLD may enable phyllosoma to disperse far offshore, into a pelagic environment that is favorable for the survival of unprotected larval-stage individuals (Strathmann *et al*. 2002). Late-stage lobster larvae (pueruli) are fast swimmers (Serfling & Ford 1975) and may utilize strong upwelling regimes to return and settle near their natal site after dispersing offshore. If this behaviour is selectively advantageous, we would expect to observe enhanced local recruitment regardless of PLD. Furthermore, local recruitment should be more pronounced at sites with strong, persistent upwelling (Fig. 6b).

The four sites with the highest proportion of kin in Baja California, Mexico, are located in areas of persistent upwelling (Zaytsev *et al*. 2003; Fig. 6a). Although upwelling was initially proposed as a mechanism for advecting recruits away from coastal areas (Roughgarden *et al*. 1988), subsequent studies have questioned that prediction (e.g. Shanks & Brink 2005; Morgan *et al*. 2009; Morgan & Fisher 2010; reviewed in Shanks & Shearman 2009). Genetic studies have proven equivocal in this regard: some have found reduced genetic substructure between populations in years with greater cumulative upwelling (e.g. Flowers *et al*. 2002; Barshis *et al*. 2011), and others have found upwelling dynamics to be insufficient to explain temporal and spatial genetic patterns (e.g. Toonen & Grosberg 2011). Passive larval dispersal models indicate that the effects of upwelling may depend on whether larvae stay near the surface (upwelling advects larvae offshore) or undergo regular migrations to depth (upwelling delivers larvae to the coast) (Byers & Pringle 2006; Marta-Almeida *et al*. 2006).

Spiny lobsters are known to have relatively large larvae capable of dynamic movement. Both the phyllosoma and puerulus stages show evidence of active movement in the pelagos, with phyllosoma exhibiting diel vertical migrations as well as horizontal movements (Kittaka 1994; Chiswell & Booth 1999; Phillips *et al*. 2006; Butler *et al*. 2011), and pueruli demonstrating rapid swimming, navigation towards the coast and habitat settlement preferences (Serfling & Ford 1975; Jeffs *et al*. 2005; Phillips *et al*. 2006). In *P. interruptus* specifically, Pringle (1986) found both geographic and depth stratification of different stages of both phyllosoma and pueruli collected during yearly larval tows in the California Current Ecosystem, suggesting active ontogenetic shifts in larval depth preference. Incorporating such larval behaviours into biophysical models of dispersal in a congeneric species, *Panulirus argus*, resulted in a 60% or greater decrease in the average distance a larva is predicted to settle from its release site compared with simulations of larvae that remain on the surface (Butler *et al*. 2011). Lobsters in the California Current Ecosystem rely on kelp forest habitat for survival: *P. interruptus* has much higher relative survival rates in the presence of kelp than in surrounding areas where kelp is absent (Mai & Hovel 2007). Given the ephemeral dynamics of kelp forest habitat (Reed *et al*. 2006) and the high variability in ocean conditions in this region, these lobsters may have evolved similarly complex behaviours as their congeners to increase successful local recruitment despite their extremely long PLD (e.g. Shanks & Eckert 2005).

## Conclusion

Here, we present a novel approach to understand contemporary drivers of population differentiation in systems with high gene flow. In isolation, the population-level data present a commonly documented scenario among marine species: no evidence for any particular regional separation or isolation-by-distance patterns, but low and significant pairwise differences among populations. While the agreement between nuclear and mitochondrial markers confirms that the results are not due to statistical artifact, the nature of *F*-statistics leaves us without a clear indication of what is driving the pattern of genetic differentiation. The addition of kinship analyses reveals how alleles are shared between individuals, rather than just among populations, and provides an independent test of the hypothesis that population genetic structure as measured by *F*-statistics is a result of population connectivity. In this case, the majority of locations contained an excess of closely related individuals. This supports the inference that either self-recruitment or some form of coordinated larval delivery is driving population-level genetic differences in a species that would be expected to be broadly dispersive throughout its range, given its extremely long PLD. In combination with regional oceanographic data and larval dispersal behaviour, kinship analyses provide evidence for a mechanism of differentiation in an otherwise murky population genetic data set. The ability to directly test hypotheses about what drives population genetic substructure in high gene-flow species by independent means, such as kinship or coalescent analyses, provides greater confidence in the underlying causes of population substructure than summary statistics alone. As the ease of developing greater numbers of genetic markers increases, individual-based analyses such as relative kinship indices can provide a valuable complement for understanding patterns in traditional population genetics data sets.

## Data accessibility

DNA sequences: GenBank accession nos: KC775458–KC775695.

Sample ID's, collection locations, microsatellite genotypes and an mtDNA COI sequence alignment: Dryad entry doi: 10.5061/dryad.j6332.

## Supporting information

**Table S1** Isolation by distance (IBD) results from Mantel tests conducted in genodive 2.0b20 to examine patterns of genetic differentiation across multiple spatial scales based on sampling locations: all sites, only California sites, only Mexico sites, only continental sites, and only island sites.

**Table S2** Locus-specific diversity indices and *F*_ST_ values as calculated in genodive for each of the seven nuclear microsatellite loci, and null allele frequency for each microsatellite locus as calculated in *ML-Relate*.

**Table S3** Comparison of *ML-Relate* calculations of relatedness (*r*) and genodive calculations of kinship for each sampling location.

**Fig. S1** Linear regression of mean pairwise local *F*_ST_ (squares, dashed line) and *D*est_Chao (circles, solid line) at each site on the combined proportion of full- and half-sibs as determined in *ML-Relate* for seven microsatellite loci.

**Fig. S2** Log-linear regression of mean relatedness (*r*) at each site on the distance (km) to the nearest edge of an area of high upwelling intensity (from Fig. 6a).

**Fig. S3** Median-joining network for *Panulirus interruptus* mtDNA, constructed using 454 base pairs of cytochrome *c* oxidase subunit I (COI) from each of 931 individuals in the program network.
