# Supplementary material for: Combined analyses of kinship and FST suggest potential drivers of chaotic genetic patchiness in high gene-flow populations
Source: Mol Ecol. 2013 Jun 26;22(13):3476–94. doi: 10.1111/mec.12341 (PMC3749441; doi:10.1111/mec.12341)
Supplement: Supplementary file 1 [file mec0022-3476-SD1.pdf]

Table S1. Isolation by distance (IBD) results from Mantel tests conducted in GenoDive 2.0b20 to examine patterns of genetic differentiation across multiple spatial scales based on sampling locations: All sites, only California sites, only Mexico sites, only continental sites, and only island sites. Data reported include the marker used (Marker), the metric used (Metric), the IBD equation (Equation), the number of sites included in the analysis (N-sites), the error sum of squares (Error SS), the total sum of squares (Total SS), the r-squared value (R^2), and the *P*-value (*P*-value). Although there is one significant comparison (*), this was not significant after correcting for multiple comparisons.

Sample Sites	Marker	Metric	Equation	N-sites	Error SS	Total SS	R^2	*P*-value
All Sites	Microsatellites	Jost D_{est_Chao}	Y = 0.051 + (-0.004 * X)	17	0.125	0.126	0.009	0.198
		F_{ST}	Y = 0.007 + (-0.000 * X)	17	0.002	0.002	0.011	0.177
	Cytochrome Oxidase I	Jost D_{est_Chao}	Y = 0.063 + (-0.006 * X)	17	1.094	1.097	0.003	0.330
		Φ_{ST}	Y = -0.002 + (0.001 * X)	17	0.014	0.014	0.012	0.154
California Sites Only	Microsatellites	Jost D_{est_Chao}	Y = 0.022 + (0.000 * X)	7	0.004	0.004	0	0.470
		F_{ST}	Y = 0.002 + (0.000 * X)	7	0	0	0.002	0.401
	Cytochrome Oxidase I	Jost D_{est_Chao}	Y = 0.052 + (0.002 * X)	7	0.168	0.168	0	0.416
		Φ_{ST}	Y = 0.006 + (0.000 * X)	7	0.001	0.001	0	0.477
Mexico Sites Only	Microsatellites	Jost D_{est_Chao}	Y = 0.071 + (-0.006 * X)	10	0.065	0.066	0.017	0.266
		F_{ST}	Y = 0.009 + (-0.001 * X)	10	0.001	0.001	0.018	0.256
	Cytochrome Oxidase I	Jost D_{est_Chao}	Y = -0.009 + (0.003 * X)	10	0.227	0.227	0.001	0.433
		Φ_{ST}	Y = -0.010 + (0.003 * X)	10	0.007	0.007	0.039	0.126
Continental Sites Only	Microsatellites	Jost D_{est_Chao}	Y = 0.070 + (-0.005 * X)	10	0.058	0.058	0.014	0.282
		F_{ST}	Y = 0.009 + (-0.001 * X)	10	0.001	0.001	0.014	0.278
	Cytochrome Oxidase I	Jost D_{est_Chao}	Y = -0.073 + (0.013 * X)	10	0.162	0.167	0.030	0.178
		Φ_{ST}	Y = -0.015 + (0.004 * X)	10	0.007	0.007	0.075	0.063
Island Sites Only	Microsatellites	Jost D_{est_Chao}	Y = 0.057 + (-0.008 * X)	7	0.004	0.005	0.094	0.108
		F_{ST}	Y = 0.008 + (-0.001 * X)	7	0	0	0.093	0.111
	Cytochrome Oxidase I	Jost D_{est_Chao}	Y = -0.050 + (0.026 * X)	7	0.301	0.306	0.017	0.300
		Φ_{ST}	Y = 0.020 + (-0.003 * X)	7	0	0	0.258	0.022*

Table S2. Locus-specific diversity indices and F_{ST} values as calculated in GenoDive for each of the seven nuclear microsatellite loci, and null allele frequency for each microsatellite locus as calculated in *ML-Relate*. Metrics included are the total number of alleles for each locus (N), effective number of alleles for each locus (A_{eff}), observed heterozygosity (Ho), within-population heterozygosity (Hs), total heterozygosity (Ht), F_{ST} (F_{ST}), significance test for F_{ST} (*P*-value (F_{ST})) based on 100,000 permutations in GenoDive, percentage of genotyping error (% Genotyping Error), percentage of null alleles (% Null Alleles), and the *P*-value from a Monte-Carlo randomization test (20,000 randomizations) of whether the null-allele frequency is significant (*P*-value (NA)).

Locus	N	A_{eff}	Ho	Hs	Ht	F_{ST}	*P*-value (F_{ST})	% Genotyping Error	% Null Alleles	*P*-value (NA)
**A110**	5	2.203	0.497	0.552	0.552	0.001	0.296	0.00%	3.69%	0.0035
**A5**	35	11.412	0.943	0.921	0.924	0.003	0.002	1.35%	0.00%	0.3612
**A102r**	37	13.084	0.875	0.933	0.935	0.003	0.010	4.05%	3.10%	0.0001
**pin244**	104	23.301	0.909	0.967	0.970	0.004	< 0.0005	2.70%	2.95%	< 0.00005
**pin29L**	75	23.036	0.842	0.967	0.969	0.002	0.002	4.05%	6.63%	< 0.00005
**pin10**	35	10.137	0.897	0.911	0.920	0.011	< 0.0005	1.35%	1.20%	0.0098
**pin189**	41	14.852	0.858	0.943	0.944	0.002	0.023	2.70%	4.60%	< 0.00005

Table S3. Comparison of *ML-Relate* calculations of relatedness (*r*) and GenoDive calculations of kinship for each sampling location. Counts of Relatedness (*r*) represent the number of individual pairwise comparisons that had the lowest log-likelihood score for each category, as calculated in *ML-Relate*: UR (most likely unrelated), HS (most likely half-sibs), FS (most likely full-sibs), PO (most likely parent-offspring). Counts of Kinship represent the number of pairwise comparisons that fell within the categories of kinship as described in the text: unrelated (UN), k < 0.047; quarter-sib (QS), 0.047 < k < 0.09375; half-sib (HS), 0.09375 < k < 0.1875; full-sib (FS), 0.1875 < k < 0.375; and nearly identical (NI), k > 0.375. The Total Number of Pairwise Comparisons is the same for both metrics. Also reported are the mean site-specific values of relatedness (*r*), as calculated by taking the mean value of all maximum likelihood estimates of (*r*) for all pairwise individual comparisons within a site (*ML-Relate* Mean (*r*)), and the mean values of the Loiselle *et al.* 1995 coancestry metric, as calculated in GenoDive (Loiselle Mean Kin). Site-specific mean (*r*) and mean kin are significantly correlated (Pearson R = 0.92, *P* < 0.0005).

	**Counts of Relatedness (*r*)**				**Counts of Kinship**					**Total Number**	***ML-Relate***	**Loiselle**
**Site**	**UR**	**HS**	**FS**	**PO**	**UR**	**QS**	**HS**	**FS**	**NI**	**of Comparisons**	**Mean (*r*)**	**Mean Kin**
**CARP**	2415	278	8	0	2125	350	212	12	2	2701	0.0364	0.0025
**SMI**	2522	312	16	0	2203	410	218	18	1	2850	0.0396	0.0035
**SCI**	1289	135	7	0	1124	213	86	8	0	1431	0.0355	0.0016
**MLBU**	2243	220	22	0	2019	312	131	18	5	2485	0.0375	0.0005
**SCAT**	2860	358	22	0	2537	449	237	17	0	3240	0.0433	0.0027
**SNI**	613	82	7	1	526	95	66	16	0	703	0.0524	0.0056
**CLEM**	267	32	1	0	232	47	19	2	0	300	0.0378	0.0022
**CRDO**	1770	174	10	0	1552	262	124	15	1	1954	0.0361	-0.0014
**PTN**	1400	167	29	0	1240	221	107	21	7	1596	0.0504	0.0078
**PBDA**	902	167	12	0	756	190	112	23	0	1081	0.0575	0.0163
**PBJ**	3882	396	93	0	3573	499	212	74	13	4371	0.0439	0.0051
**IGP**	2092	241	13	0	1860	338	142	6	0	2346	0.0404	0.0010
**ODL**	782	77	2	0	717	95	49	0	0	861	0.0336	-0.0013
**PE**	858	128	4	0	777	136	72	5	0	990	0.0417	0.0037
**BTG**	668	97	15	0	583	105	74	13	5	780	0.0543	0.0112
**ABRE**	1910	227	8	0	1728	268	139	10	0	2145	0.0391	0.0006
**BMG**	960	118	3	0	907	133	41	0	0	1081	0.0352	0.0005

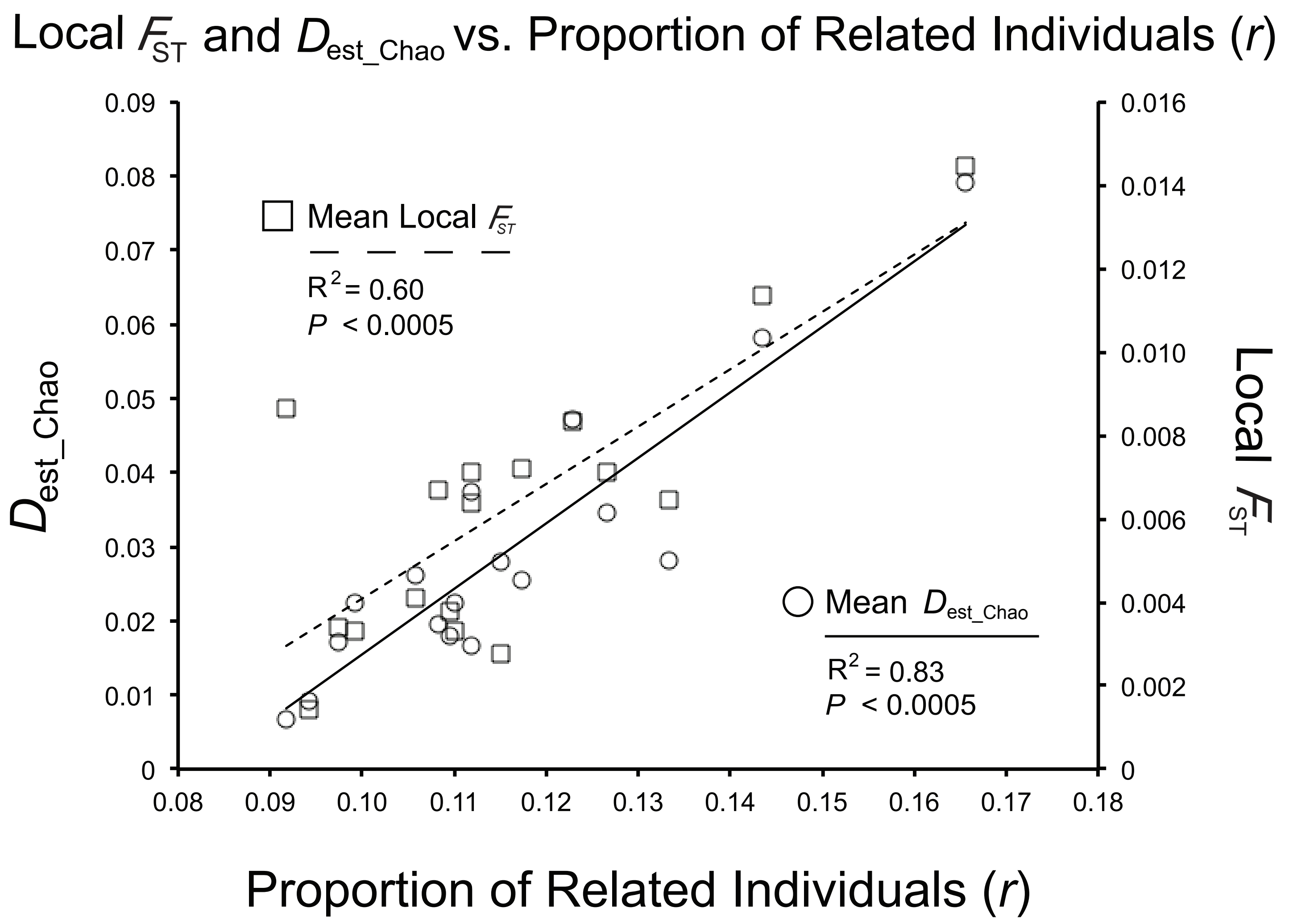

Fig. S1 Linear regression of mean pairwise local F_{ST} (squares, dashed line) and D_{est_Chao} (circles, solid line) at each site for seven microsatellite loci. Both metrics were regressed on the proportion of combined within-site half (r = 0.25) and full (r = 0.50) sibs as calculated using a maximum likelihood estimation of relatedness (r) in *ML-Relate*. Only comparisons where half or full sibs were determined to be both a significant (P < 0.05) and the most likely relationship were included. The linear regression of F_{ST} versus proportion of related individuals, though not shown here, is also significant (R^2 = 0.82, P < 0.0005).

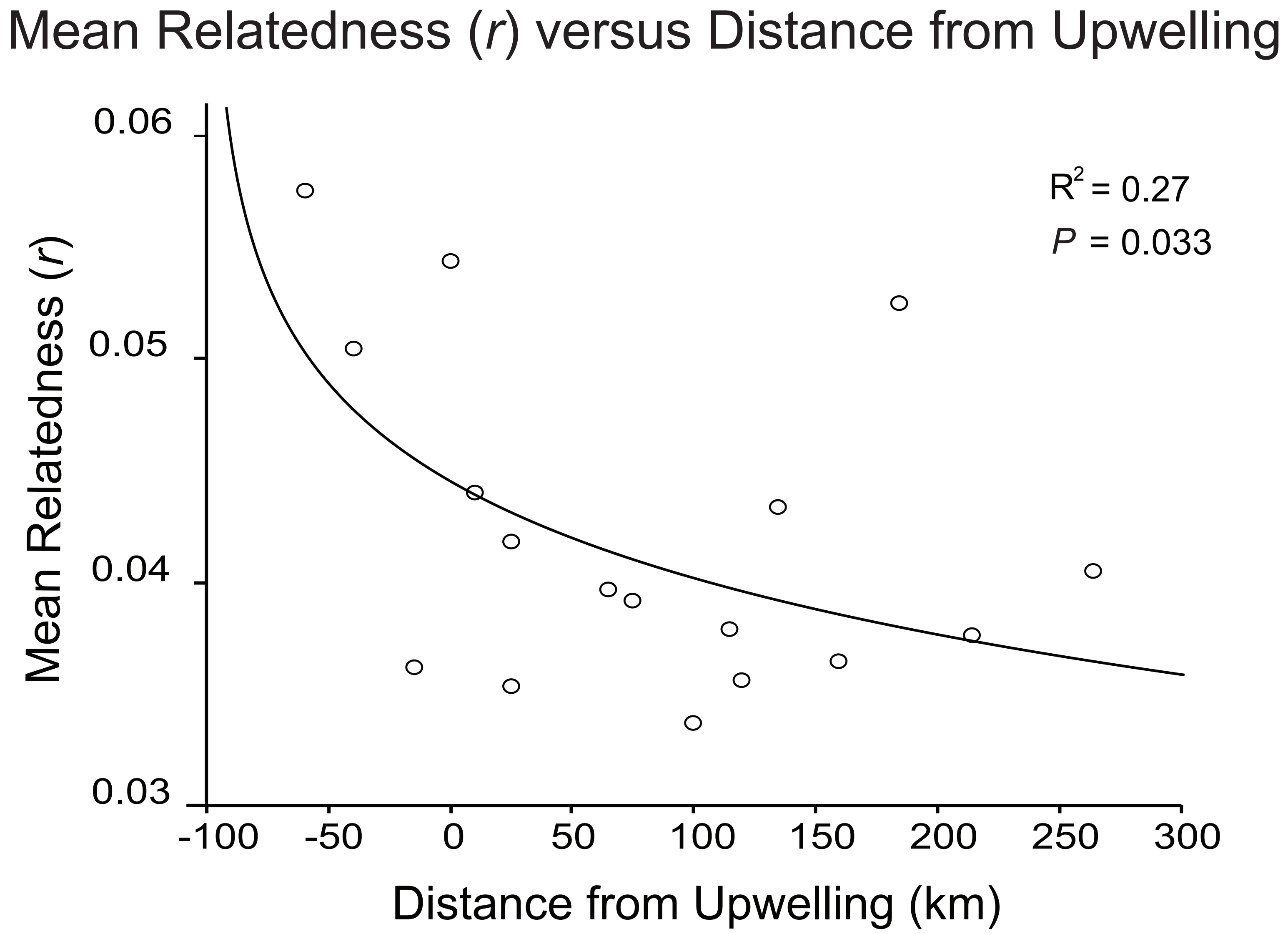

Fig. S2 Log-linear regression of mean relatedness (*r*) at each site on the distance (km) to the nearest edge of an area of high upwelling intensity (from (Fig.6a)).

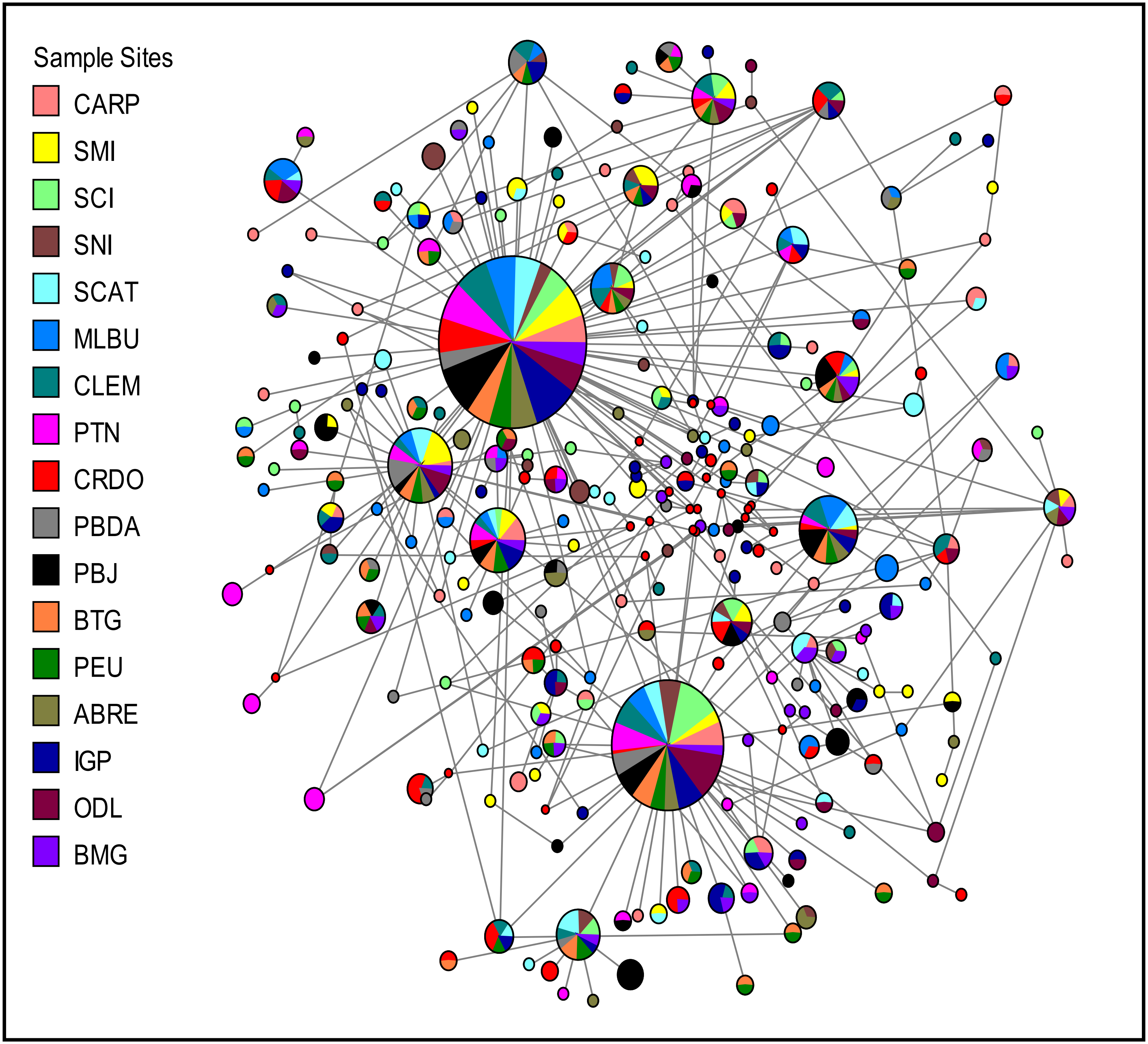

Fig. S3 Median-joining network for *Panulirus interruptus* mtDNA, constructed using 454 base pairs of cytochrome *c* oxidase subunit I (COI) from each of 931 individuals in the program NETWORK. Each circle is a unique haplotype proportional in size to the number of individuals with that haplotype. The two largest circles represent 235 and 95 individuals. The smallest circle represents one individual. Colors correspond to one of 17 locations where the individual haplotypes were found (see key, Fig. 1, Table 1). Lines here are not representative of distance between haplotypes, although most haplotypes only differ by one base pair (see Fig. 2).
